# Experimental Investigation of the Effects of Loss of Blood

**Published:** 1833-04-01

**Authors:** 


### Experimental Investigation of the Effects of Loss of Blood. By *Marshall Hall*, M.D. F.R.S. &c.

[Med.-Chir. Trans.]

Dr. Hall, after observing as accurately as possible the effects of hæmorrhage and bloodletting on the human subject, has very laudably turned his attention to experiments on animals, in order more fully to investigate this important inquiry. His objects were, first, to ascertain the effects of loss of blood in health—2dly, in the different epochs of life—3d, the organic changes that resulted from loss of the vital fluid—4th, to fix the rules and limits of sanguineous depletion as a remedial agent. The present paper embraces the subjects of syncope—excessive reaction—gradual sinking—immediate dissolution—the organic changes.

I. Syncope.

This phenomenon is much modified by position, while blood is flowing. It appears to depend on sudden abstraction of blood from the *brain*, according to our author, and the following are his reasons for so thinking.

"The languor of the eye and of the expression; the panting, or sighing, observed in the breathing; the cessation or extreme diminution of the frequency and force of the action of the heart and of the arteries; the loss of appetite; the nausea and vomiting; the debility of the voluntary muscles and the relaxation of the sphincters of the bladder and rectum, all appear to have this origin.

The truth of this remark, in regard to some of these phenomena, is obvious from the influence and effects of posture. The most remarkable of the phenomena of syncope may be renewed, after they have disappeared, by placing the animal in such a posture that the head shall be as much elevated, and the other parts of the body as much depressed, as possible: the countenance and the eye languish, and the head droops; the mouth then opens and the respiration becomes panting; if at this moment the ear be applied over the region of the heart, its action, distinctly perceived before, *is found to be either suspended, or so feeble as scarcely to be heard.* On reversing the position, the animal is immediately relieved, it raises its head and looks about, the respiration, although impeded by so constrained a position, is greatly restored, and the beat of the heart *becomes instantly perceptible,* and in a short time loud and strong." 253.

Now we are of opinion, that the whole of the phenomena of fainting depend, not on the abstraction of blood from the *brain*, a thing which cannot, in fact, be done, but on the inability of the heart itself to continue the circulation, and thus to supply the brain and all other parts with a *current* of blood. The effects of position on the heart itself are more easily explicable than on the brain. When the whole body is horizontal, the heart circulates the blood more easily than when any part, and especially so large a part as the head, is elevated. It is well known that the heart will continue to act for some time after the brain is removed—but no one ever saw the sensorial functions continue a single minute after the heart had ceased to move. The pulse may not be perceptible, though the heart is acting; but we need not dwell on this part of the subject farther.

II. Excessive Re-action.

The animal soon recovers from the milder degrees of syncope, the heart regaining, but not exceeding its usual force; but if the detraction of blood be repeated at such intervals, and in such quantities, as not to endanger life, the re-action passes the limit of healthy function, and becomes excessive, presenting some curious and important phenomena. Here our worthy author appears to feel the error of attributing syncope to affection of the brain, since he is forced to allow that the reverse, or rather the effect of that state, "is obviously an affection of the *heart*." We are confident that the syncope is just as much a failure of the heart's action, as re-action is an abnormal condition of the function. The author goes to entangle himself and his readers with reasons for this difference of seat in the cause of syncope and re-action, and, of course, makes confusion more confounded. In his anxiety, however, to prove his position, he has overshot the mark, and has fallen into an erroneous exposition of visible phenomena. During re-action, Dr. Hall tells us that, although the head is seen and felt to throb with the violence of the circulation, "the brain does not appear to be at all affected." On this point we join issue with Dr. H.; and we maintain from careful observation and personal feeling, that in all kinds of re-action, from that which succeeds a cold bath up to that which follows the cold stage of ague, the sensorial functions are affected in proportion to the strength of that re-action. Dr. Hall himself tells us that, in the re-action after bleeding or hæmorrhage, "the eye is bright, the countenance and manner lively." Are these signs that the sensorium is unaffected?—Are they not the reverse of the sensorial phenomena presented in syncope, or the approach to syncope? But Dr. Hall has given us but a niggardly account of the phenomena. The sensibility of all the organs of sense is augmented during the re-action—thought itself is quickened—imagination is exalted—temperature is raised—in short, every function dependent on the brain and nerves is stimulated and elevated above par, in proportion to the re-action. For the truth of this statement, we appeal to every one who has observed or felt —to every one who chooses to observe in future at the bed-side.

### III. Sinking.

Syncope may be easily induced by the detraction of blood, and re-action may be, without difficulty, subdued again. The state termed "sinking" is not easily induced in animals. If the detraction of blood be moderate, the animal soon rallies—if excessive, dissolution takes place, and not gradual sinking:—The animal dies during the night. In one or two of the experiments, however, there was something like an approach to the state of sinking observed in the human subject.

### IV. Dissolution.

This, our author thinks, is principally an affection of the brain, like syncope. We are disposed to think that *dissolution* indicates a pretty considerable affection of various organs and functions including the heart among the rest. The experiments, however, shewed that the heart often continued to act, after the functions of the brain appeared to cease.

"After the last gasp, after the last convulsive movement, the heart can be seen, felt, and heard beating with force, and the pulse of the femoral artery is quite distinct. We observed this in several instances, and in one for many minutes." 261.

### V. Organic Changes.

If an animal die from the effects of one, two, or three abstractions of blood, the brain and other viscera "do not appear to be remarkably blanched;" but, if existence has been more protracted under the influence of loss of blood, the said organs appeared blanched. This circumstance, Dr. H. thinks, militates against the doctrines of Kellie and Sanders; but we apprehend that these experiments were chiefly on animals bled to death at once, or at very few bleedings, in which cases the head was found not only not blanched, but even unusually gorged with blood.

In respect to the lungs, it was only in two experiments that any disorganizations were found. In these there were traces of emphysema, congestion, or hepatization, with fluid in the bronchia and air-cells. The right side of the heart generally contained blood—the spleen was small—other viscera, except the liver, blanched.

### VI. The Blood.

When separated into serum and crassamentum, the proportion of the latter at first exceeded that of the former; but this proportion was gradually inverted—the quantity of serum augmenting, whilst that of the crassamentum diminished. In many cases, a creamy substance floated on the serum, consisting of oily matter soluble in æther, probably fat absorbed. In only one instance was there an appearance of buff.

In respect to the pulse, it was sometimes rendered slower, sometimes quicker, by the loss of blood. As to the capillary circulation, the conjunctiva becomes blanched—the ears and feet cold.

### Practical Applications.

"We may deduce from the foregoing experiments the extreme difficulty of ascertaining the state of immediate danger arising from loss of blood, whilst the animal retains the horizontal position. There is no phenomenon which clearly indicates such danger.

In the prosecution of these experiments I had continually to regret the want of a criterion for the extent to which I might allow the blood to flow,—a criterion which is afforded to the veterinarian by the position of the horse, and of which the physician may avail himself by directing his patient to be placed upright. The dog is apt to pass from the sitting to the prone position, and then more blood may be withdrawn than he can bear to lose, before any distinct criterion of the sufficient quantity was apparent.

This difficulty was particularly felt when we attempted to induce the slowly sinking state. If we took too little blood, the animal rallied perhaps completely; if too much, it sank immediately. In another position the signs of an incipient syncope would have afforded us the guide and the warning which were wanted.

From observations made on the human subject, and in the course of these experiments, I am alike persuaded that bloodletting ought never to be employed in the horizontal position. The quantity of blood taken, although apparently moderate, may be beyond the powers of the system to bear. But if the erect position be adopted, the occurrence of syncope limits the flow of blood, and the change to the horizontal position affords a prompt refuge from danger.

We learn from these experiments, that during the state of excessive re-action there is danger of effusion into the brain. That state must therefore be watched with peculiar anxiety in the cases of our patients.

We learn also that there is great danger of mistaking the state of excessive re-action for diseases of the heart and its valves. A correct diagnosis is, of course, of the utmost moment." 267.

The experiments themselves we must pass over; and with the exception of the point of physiological difference between us, we beg to express our high sense of the talented author's merit in this and other papers.

